# Chronic Kidney Disease as a Predictor of Mortality in Hospitalized Inflammatory Bowel Disease Patients With Clostridioides difficile Infection

**DOI:** 10.7759/cureus.88113

**Published:** 2025-07-16

**Authors:** Joelle Sleiman, Andrew Gaballa, Taimur Aslam, Taqi A Rizvi, Suzanne El Sayegh

**Affiliations:** 1 Internal Medicine, Staten Island University Hospital, New York City, USA; 2 Internal Medicine, State University of New York Downstate Health Sciences University, New York City, USA; 3 Nephrology, Staten Island University Hospital, New York City, USA

**Keywords:** cdiff, end stage renal disease (esrd), (ibd) inflammatory bowel disease, inpatient mortality rate, kidney disease

## Abstract

Introduction

Clostridioides difficile infection (CDI) leads to significant morbidity and mortality in hospitalized patients. We aim to investigate whether chronic kidney disease (CKD) or end-stage renal disease (ESRD) are predictors of mortality in admitted patients with CDI, and whether the presence of inflammatory bowel disease (IBD) has any impact on the mortality rate.

Methods

The data of 133,099 hospitalized patients with CDI were analyzed from the National Inpatient Sample (NIS) database from 2016 to 2018. Baseline risk factors were identified using ICD-10 codes. Propensity score matching was used to match CKD/ESRD patients with patients without kidney disease based on age, gender, and IBD status (Crohn's disease or ulcerative colitis). A multivariable logistic regression model was used to establish the relationship between variables and adjusted for underlying risk factors. The primary endpoint was all-cause mortality among hospitalized patients with CDI, particularly those with IBD.

Results

Our cohort included 133,099 hospitalized patients with CDI, among whom 20,700 (15.6%) had CKD, 12,178 (9.1%) had ESRD, and 6104 (4.6%) had IBD. The mean age was 74, 63, and 63 years (p<0.001) in patients with CKD, ESRD, and those without kidney disease, respectively. CDI patients with ESRD had a higher proportion of males (6095, 50.3%), compared to those with CKD and without kidney disease (9494, 45.9%, and 40,876, 40.8%, respectively; p<0.001). Propensity score matching was performed, and the patients were matched on age, resulting in a 1:1 exact match for 32,878 patients. Logistic regression analysis showed that in CDI patients with IBD, CKD was associated with a statistically insignificant trend towards a higher all-cause mortality rate as compared to CDI patients without IBD (odds ratio, 1.682 vs odds ratio, 1.229, p>0.05). However, ESRD patients had a significantly higher all-cause mortality rate (Odds ratio, 3.738, 95% CI 2.175-6.425) among hospitalized CDI patients with IBD compared to those without IBD (odds ratio, 1.612, 95% CI 1.486-1.749) (p<0.05).

Conclusion

Kidney disease, particularly ESRD, is associated with a significant increase in mortality in CDI patients with IBD compared to those without IBD. These findings highlight the importance of aggressive CDI management in patients with IBD and ESRD.

## Introduction

*Clostridioides difficile* (*C. difficile*) is a gram-positive enteric bacterium that spreads through the fecal-oral route and causes *Clostridioides difficile* infection (CDI) [1]. CDI may be asymptomatic or may present with diarrhea, pseudomembranous colitis, or toxic megacolon [2]. It can be a community-acquired or healthcare-associated infection. It is the most common healthcare-associated infection in the US, and its incidence is increasing [3-5]. It is also associated with significant morbidity, mortality, and healthcare-associated financial burdens, totaling approximately $1.5 billion annually in the United States. Mortality rates range between 5% and 10%, with significantly higher rates in patients who are elderly, critically ill, or have multiple comorbidities [6,7].

The most common risk factors for CDI in the general population are old age, antibiotic use, hospital admission, and underlying comorbid conditions, including kidney disease. It has been shown that chronic kidney disease (CKD) or end-stage renal disease (ESRD) increases the risk, severity, morbidity, and mortality of CDI in the general population due to multiple intersecting factors [8,9]. These include frequent antibiotic use, repeated hospitalizations, and profound immune dysregulation. Specifically, uremia-related alterations impair both innate and adaptive immunity, such as reduced neutrophil chemotaxis, phagocytic dysfunction, and T-lymphocyte abnormalities, which diminish host defense mechanisms against pathogens like *C. difficile* [10]. Furthermore, this detrimental effect is even greater in patients with ESRD than in those with CKD [11,12]. Similarly, patients with inflammatory bowel disease (IBD) are at higher risk of developing CDI, with increased disease severity, high morbidity, and mortality as compared to the general population [13,14] The overlapping risk factors in IBD include chronic inflammation, use of broad-spectrum antibiotics, corticosteroids, immunosuppressive therapies, frequent healthcare exposure, and impaired nutritional status. Nutritional deficiencies, including hypoalbuminemia and micronutrient depletion, can compromise intestinal mucosal integrity and immune responses, increasing susceptibility to CDI [15,16]. Certain subgroups, such as older adults, females, patients with Ulcerative Colitis (UC), and those on biologics or NSAIDs, are at even greater risk [17]. Despite these shared pathways, the influence of kidney disease on CDI-related outcomes in IBD patients remains underexplored. To date, no large retrospective study has quantified whether CKD or ESRD independently increases CDI-related mortality among hospitalized patients with IBD. Understanding this relationship is critical, as individuals affected by both IBD and kidney disease may require tailored clinical protocols to mitigate the risk of severe CDI and associated mortality. Our study aims to address this gap by examining the impact of CKD and ESRD on CDI-related mortality in hospitalized patients with IBD, compared to the general population. Identifying high-risk groups can guide more aggressive therapeutic strategies and improve patient outcomes.

This article was previously presented as a meeting abstract at the 2024 ACG Annual Scientific Meeting on October 29th, 2024.

## Materials and methods

Study design and population

This retrospective observational cohort study included 133,039 hospitalized patients aged ≥18 years with a confirmed diagnosis of *Clostridioides difficile* infection (CDI), identified using specific ICD-10 codes from the National Inpatient Sample (NIS) database between 2016 and 2018. The diagnosis of CDI and baseline patient characteristics, comorbid conditions including chronic kidney disease (CKD) and end-stage renal disease (ESRD), and inflammatory bowel disease (IBD) status (Crohn's disease or ulcerative colitis) were identified using the International Classification of Diseases, Tenth Revision (ICD-10) codes. Institutional Review Board approval was not required because NIS data is de-identified and publicly available.

Inclusion and exclusion criteria 

We included all adult patients (≥18 years) hospitalized with a primary or secondary diagnosis of CDI during the study period (2016-2018), as identified using ICD-10 codes. Patients with missing demographic data (age, gender), unknown discharge status, or incomplete hospitalization records were excluded from the analysis.

Statistical analysis

Propensity score matching was performed to control for confounding variables when comparing CDI patients with CKD and ESRD to those without kidney disease. 1:1 exact matching was based on age, gender, and IBD status (Crohn's disease or ulcerative colitis). Exact matching was used to ensure close comparability between groups. Following matching, a multivariable logistic regression model was used to examine the association between CKD/ESRD and mortality in CDI patients, adjusting for relevant baseline characteristics. The primary endpoint was all-cause mortality among hospitalized patients with CDI, particularly those with IBD.

The data were analyzed using Statistical Package for the Social Sciences (IBM Inc., Armonk, New York) software, with categorical variables being analyzed by way of the chi-square test and reported as frequencies, whereas continuous variables were analyzed using the ANOVA test and reported as means. A p-value of <0.05 was considered statistically significant.

## Results

Our cohort included 133,099 hospitalized patients with CDI, among whom 20,700 (15.6%) had CKD, 12,118 (9.1%) had ESRD, and 6,282 (4.7%) had a diagnosis of IBD. Patients with CKD were older, with a mean age of 74 years, compared to 63 years in both the ESRD and non-kidney disease groups (p<0.001). The sex distribution also varied significantly across groups. The proportion of male patients was highest in the ESRD group (6095, 50.3%), followed by those with CKD (9494, 45.9%), and lowest among patients without kidney disease (40,876, 40.8%). The baseline characteristics of the cohort are detailed in Table 1. The primary endpoint, which is all-cause mortality, occurred in 8042 (6%) of the total population.

**Table 1:** Baseline characteristics of the population Continuous variables (age) were compared using one-way ANOVA and reported as means. Categorical variables (sex, IBD status) were compared using the Chi-squared test and are reported as counts and percentages. A p-value < 0.05 was considered statistically significant. IBD - inflammatory bowel disease; CKD - chronic kidney disease; ESRD - end-stage renal disease; ANOVA - analysis of variance

Variables	No kidney disease	CKD	ESRD	p-value	Test statistic
	(N=100,221)	(N=20,700)	(N=12,118)		
Age in years, mean	63	74	63	<0.001	F = 10574.93
						Female, n (%)	59345 (59.2%)	11206 (54.1%)	6023 (49.7%)	<0.001	χ² = 517.87
Male, n (%)	40876 (40.8%)	9494 (45.9%)	6095 (50.3%)	<0.001	χ² = 517.87						
						IBD, n (%)	5584 (5.6%)	520 (2.5%)	178 (1.5%)	<0.001	χ² = 670.58

The prevalence of IBD was significantly different across the three groups, with 5,584 (5.6%) patients in the non-kidney disease group, 250 (2.5%) patients among those with CKD, and 178 (1.5%) among ESRD patients (p<0.001). Propensity score matching based on age, gender, and IBD status yielded exact matches in 32,878 patients. Logistic regression analysis based on IBD diagnosis showed that in CDI patients with IBD, CKD was associated with a trend towards a higher all-cause mortality rate compared to CDI patients without IBD; however, this was not statistically significant (odds ratio (OR) 1.682 vs odds ratio 1.229, p>0.05) (Table 2). 

**Table 2:** Logistic Regression Analysis Based on IBD Diagnosis Odds Ratios (OR) were calculated using logistic regression to assess the association between kidney disease and in-hospital mortality in patients with Clostridioides difficile infection (CDI), stratified by the presence or absence of IBD. The reference group for each model was patients without kidney disease (i.e., neither CKD nor ESRD). CI - confidence interval; CKD - chronic kidney disease; ESRD - end-stage renal disease; IBD - inflammatory bowel disease; OR - odds ratio

Patients without an IBD diagnosis			
Variable	OR	95% CI	p-value
CKD	1.229	(1.142-1.322)	< 0.001
ESRD	1.749	(1.486-1.612)	< 0.001
Patients with an IBD diagnosis			
Variable	OR	95% CI	p-value
CKD	1.682	(1.075 -2.630)	0.023
ESRD	3.738	(2.175 -6.425)	< 0.001

Whereas patients with ESRD had a significantly higher all-cause mortality rate (odds ratio 3.738, 95% CI 2.175-6.425) among hospitalized CDI patients with IBD compared to those without IBD (odds ratio 1.612, 95% CI 1.486-1.749) (p<0.05) (Table 2).

**Figure 1:**
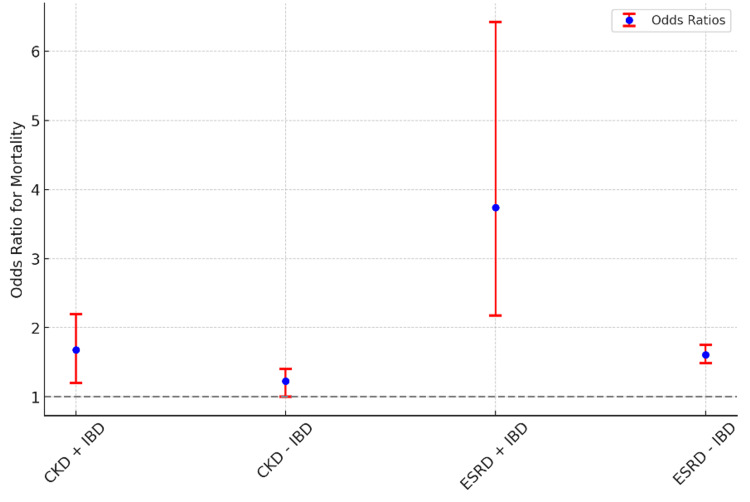
Odds ratios for in-hospital mortality among CDI patients by kidney disease and IBD status The figure displays odds ratios (blue dots) and their corresponding 95% confidence intervals (red lines) for in-hospital mortality among patients with CDI, stratified by the presence or absence of CKD, ESRD, and IBD. The horizontal dashed line represents OR = 1, indicating no association with mortality. CDI - *Clostridioides difficile* infection; CKD - chronic kidney disease; ESRD - end-stage renal disease; IBD - inflammatory bowel disease

These findings are graphically illustrated in Figure 1, which compares the odds ratios for mortality among CKD and ESRD patients with and without IBD, highlighting the disproportionately higher mortality risk among patients with ESRD and coexisting IBD, who demonstrated significantly increased odds of death.

## Discussion

Our findings indicate that kidney disease is associated with significantly higher CDI-related mortality in patients with IBD compared to those without IBD. Specifically, among patients with IBD, CKD increased in-hospital mortality by 68% (OR 1.682, 95% CI 1.075-2.630, p=0.023), and ESRD increased it by 270% (OR 3.738, 95% CI 2.175-6.425, p<0.001). In contrast, in patients without IBD, CKD and ESRD increased mortality by 23% (OR 1.229) and 61% (OR 1.749), respectively. These results emphasize the importance of early and aggressive therapy in patients with IBD and concomitant kidney disease, especially ESRD, as they have a very high inpatient mortality rate.

This aligns with prior studies showing that patients with CKD or ESRD experience immune dysfunction, which increases morbidity and the risk of recurrent CDI, especially in ESRD patients [11,12,18]. In our cohort, the observed increase in mortality may reflect the synergistic impact of immunocompromise and systemic inflammation in patients with both renal impairment and IBD.

In patients with IBD, research has demonstrated that IBD can independently increase the risk of CDI in hospitalized patients, and the risk is double in patients with ulcerative colitis (UC), likely due to UC's colonic involvement, which overlaps with the primary site of CDI [19,20]. Data have also demonstrated that IBD is associated with higher mortality and poorer outcomes in patients with CDI [21]. Multiple mechanisms in IBD pathophysiology can explain the higher risk of CDI in this population. Disruption of gut microbiota, along with the chronic inflammatory state and immune dysfunction associated with IBD, increases the risk of infections, including CDI and its complications [22,23]. Patients with IBD are frequently treated with immunosuppressive therapies, which increase their vulnerability to CDI. Additionally, the use of antibiotics further elevates the risk by disrupting the gut microbiota, reducing colonization resistance, and promoting *C. difficile* overgrowth [24]. 

Emerging data indicate that CKD and ESRD are increasingly prevalent among patients with IBD, affecting approximately 4-9% and up to 0.5% of this population, respectively. This highlights the importance of investigating how the coexistence of IBD and kidney disease influences disease prevalence, outcomes, and mortality, given the frequency with which this overlap is encountered in clinical practice [25,26]. Recent studies have also evaluated renal involvement in patients with IBD, showing a higher prevalence of CKD and ESRD, with IgA nephropathy, diabetic nephropathy, and interstitial nephritis being among the most common findings on kidney biopsy [27,28]. 

The effect of kidney disease on the risk of developing CDI and its severity and mortality, specifically in IBD patients, is not well studied. Due to limited existing literature, our findings significantly emphasize the importance of increased clinical attention to this high-risk population. When we studied the impact of kidney disease on CDI-associated mortality, specifically in BD patients, our results showed that CKD increases mortality by 68%, while ESRD increases mortality by 270% (compared to 23% and 61% in patients without IBD).

This increase in mortality could be explained by the fact that both patients with IBD and ESRD have increased gastrointestinal permeability, which can lead to translocation of gut microbiota, including C diff toxins, which further increases the risk of CDI and its complications [29,30]. In ESRD, uremia has been shown to impair gut barrier integrity and promote systemic endotoxemia. Similarly, IBD is associated with chronic inflammation and epithelial barrier disruption, further increasing susceptibility to microbial translocation and severe CDI-related complications. Both conditions are also prone to immune dysregulation and immunosuppression, higher exposure to antibiotics, and higher hospitalization rates, which could also contribute to worse outcomes and higher mortality of CDI. 

Multiple strategies should be implemented to prevent CDI-associated morbidity and mortality in this high-risk patient population. These interventions include strict infection control measures, avoiding predisposing factors for CDI, and minimizing the use of antibiotics [31,32]. Some studies have also recommended the use of probiotics to prevent antibiotic-associated CDI [33]. Patients with active infections should be treated aggressively with appropriate antibiotics, favoring fidaxomicin, which shows a lower recurrence rate than vancomycin in patients with IBD or kidney disease [17,34,35]. The use of fecal microbiota transplantation (FMT) has also been effective for recurrent CDI [36]. Research also supports the use of bezlotoxumab as an effective adjunctive treatment in preventing recurrent CDI, particularly in vulnerable populations, which may be considered in this group [37]. In this high-risk population, there should be a low threshold for surgical consultation if there is a high suspicion of toxic megacolon [38]. New data suggest a preventive vaccine that may protect against fatal CDI in high-risk populations, including patients with concurrent kidney disease and IBD [39,40]. Future prospective studies should evaluate the efficacy of such novel preventive strategies, including vaccination and FMT, specifically targeting patients with coexisting ESRD and IBD.

Limitations

Like any other study, our research has some limitations. The reliance on ICD-10 coding may introduce misclassification bias, which could result in either underestimation or overestimation of CDI, CKD, ESRD, or IBD diagnoses. The study is observational in nature, making it difficult to determine causality. The study is also unable to determine the severity of IBD and the specific immunosuppressive medication used by these patients, which could have influenced the outcomes of CDI. Finally, residual confounding may still be present despite propensity score matching and logistic regression analysis.

## Conclusions

In conclusion, we demonstrated that kidney disease, particularly ESRD, is associated with a significant increase in CDI-associated mortality in patients with IBD compared to those without IBD. These findings highlight the importance of aggressive CDI management in patients with IBD and CKD/ESRD. Additional research is needed to better understand the underlying mechanisms responsible for high morbidity and mortality in this patient population. Further studies and clinical trials are essential to assess the effectiveness of various CDI management strategies in patients with IBD and kidney disease, a population at significantly higher mortality, to improve survival rates.
